# Multiparameter growth-performance monitoring of Holstein dairy heifers fed on moderate- or high-energy feeding plans from birth to puberty

**DOI:** 10.1371/journal.pone.0314015

**Published:** 2024-11-21

**Authors:** Clément Colas, Eric Venturi, Marine Saget, Ludovic Métivier, Eric Briant, Mickaël Dupont, David Georget, Mathilde Daudon, Christelle Ramé, Frédéric Elleboudt, Ludovic Ducrocq, Cédric Ravineau, Pascal Salvetti, Rozenn Dalbies-Tran, Joëlle Dupont, Christophe Staub

**Affiliations:** 1 INRAE Centre Val de Loire, UE1297 Physiologie Animale de l’Orfrasière, Nouzilly, France; 2 CNRS, IFCE, INRAE, Université de Tours, UMR0085 Physiologie de la Reproduction et des Comportements, Nouzilly, France; 3 BONILAIT PROTEINES, Chasseneuil du Poitou, France; 4 TELLUS, Saint-Germain-de-Salles, France; 5 ELIANCE Station de phénotypage, Le Perroi, Nouzilly, France; Michigan State University, UNITED STATES OF AMERICA

## Abstract

Today, dairy cattle farmers are seeking to optimize expenditure and productivity throughout the lives of their animals by focusing on efficiency at all levels. One strategy for bringing forward the start of a dairy cow’s profitability phase is to advance the onset of puberty and reduce the animal’s age at their first calving. Thus, one objective of this study was to feed two groups of Holstein dairy heifers the same diet but in different quantities, with the aim of generating a growth differential of at least 200 g/day between the two groups. Thirty-eight heifer calves were followed from birth through puberty using body morphometric measurements and quantitative data collected by automatic feeders, which enabled the monitoring of both feed intake and growth for individual heifers. Routine ultrasonography was used to examine changes in the muscle and adipose tissue compartments, and x-ray tomography was used to monitor bone mineralization and rumen development. At 12 weeks of age, heifers in the optimized feeding (OPT) group had greater cortical bone thickness in the tibia compared with the control (CON) group. At 18 weeks of age, OPT heifers also had more trabecular cancellous bone. In contrast, the ruminal volumes of the heifers in the CON group were greater than those of the OPT heifers at 12 weeks. The OPT heifers had greater indices of general, skeletal, and muscular development at 9 weeks, 6 months, and 12 months. Among the circulating plasma indicators measured in this study, non-esterified fatty acids were highest in the CON calves at 6 and 12 months of age and at the onset of puberty, whereas IGF1 was highest in the OPT calves at all ages. The age at puberty of the OPT calves was 8.4 ± 0.6 months compared with 11.2 ± 1.1 months for the CON calves. The OPT heifers had an average daily weight gain of 1018 g/day from birth to the onset of puberty, which enabled them to achieve increased body fattening and to reach puberty 3 months earlier compared with the CON heifers; the average daily weight gain of the CON heifers was 780 g/day over the same period. Taken together, this study defines new reference values for a multitude of morphometric indicators, thereby enabling precise monitoring of the growth of Holstein heifers from birth to puberty.

## Introduction

The “Farm to Fork” strategy is part of the European “Green Deal” that is aiming to make food systems fair, healthy, and environmentally-friendly, and to make Europe climate-neutral by 2050. To achieve this ambitious goal, French dairy cattle farms are focusing on efficiency at all levels with the aim of optimizing their expenditure and productivity throughout the lives of their animals. As the productive phase of a dairy cow follows their first calving, the entire initial-growth phase of heifers from birth to their first calving is referred to as the non-productive phase. During this non-productive phase, the animals represent a net expense for the farmer. However, this period appears to be a key element for later productivity. Many studies have quantified the cost of rearing replacement heifers from birth to first calving, and results from such studies have varied slightly according to the time, the country in which the study was performed, and the breeding system that was used [1–4]. Overall, the costs of raising and breeding of a heifer equate to, on average, half of what the farmer will recover as milk revenue during the heifer’s first lactation period [5]. Moreover, as French dairy herds today have a first calving at 28 months, a calving interval of 12 months, and an average number of lactations per cow of 2.4, the duration of a heifer’s non-productive phase is equivalent to half of the animal’s productive lifetime [5].

One strategy for bringing forward the start of a dairy cow’s productive phase is to enable them to calve at a younger age. In fact, there seems to be a consensus in favor of changing to a first-calving age of 24 months so as to optimize farm profitability [6, 7]. The idea of reducing the average age at first calving is a concept that was proposed almost 40 years ago [8]. Apart from any economic considerations, an age of 24 months at first calving requires breeding to take place at 15 months of age; this is the time when a heifer has the best chance of conceiving [9] given that it has reached puberty at 12 to 13 months and been able to complete at least three ovarian cycles before breeding [8, 10]. However, the average age of heifers at first calving is still around 28 months in France [5], indicating that there is room for improvement.

In addition to reducing the length of the non-productive period for a heifer, early calving will facilitate herd management by reducing the number of animals required to sustain herd size, and it will also allow more rapid introduction of genetic improvements into the herd. Earlier calving also makes it possible to carry out standard operating procedures and embryo transfers more quickly, which would contribute to the dissemination of genetic progress within farms and also between farms through breeding schemes.

Accelerated growth also has an impact on the long-term careers of dairy heifers. Many studies have already shown the effects of different growth trajectories during the non-productive phase—particularly during the early preweaning phase—on the lifelong performance of dairy cows [11–19]. Two main criteria must be taken into account when assessing the success of the careers of dairy cows: milk production (both quantity and quality) and reproduction. These two activities determine the profitability of the animal and, therefore, the perennity of the herd.

A heifer’s age at first calving depends directly on their age at breeding, which in turn depends on their age at the onset of puberty. Puberty is a physiological event that occurs when a heifer has undergone sufficient development. In dairy cattle, the onset of puberty occurs at 9 to 11 months of age, and at an average body weight of 250 to 280 kg [20], which is 40% of a heifer’s adult weight (average ranges from 625 to 700 kg) [11, 21]. A fast growth rate [22], especially during the postweaning period [23], is now known to be associated with an early onset of puberty [7, 24]. The following weight targets at key ages have been established to enable successful calving at 24 months: 200 kg at 6 months, 400 kg at 15 months (the time of insemination), and 600 kg at 24 months (parturition) [25]. Farmers who want to reduce the age at which heifers reach puberty are choosing to follow optimized feeding plans to accelerate the development of their heifers. Two periods during calf rearing are particularly risky in terms of a heifer’s growth and health status: the immune gap in the first 2 weeks after birth and weaning, which is when a cow goes from receiving liquid feed to receiving solid feed. This feed-transition period must be closely managed to optimize calf growth; a minimum average daily weight gain of 800 g/day during the first 6 months of life is required to achieve early calving at 24 months [26, 27]. Does a nutrition plan generating growth at 800 g/day allow optimal expression of the heifers’ genetic potential? In France today, the nutrition plan for dairy calves is not adjusted to the genetic potential of the animals; it is instead calculated to limit the rearing expenses of an animal in its non-productive period. As numerous studies have reported that milk production can be influenced by the prepubertal development of heifers [24, 28, 29], we must ask the following: is saving money on a calf’s nutrition limiting their lifetime productivity?

In this study, we wanted to go one step further to achieve a high level of growth of above 1000 g/day in heifers during the birth to puberty period. For animals to reach this growth rate, the feeding and rearing programs being used must be very well designed. A wide range of variables to must be controlled to optimize a feeding plan, including the nature of the feed, its composition, and its availability. In particular, during the period of milk feeding, the number of meals per day, the duration of feeding, and the quality of milk are each important. All of the feeding and rearing criteria are effectors for obtaining controlled and optimized growth.

For a long time, farmers have been advised against increasing the amount of milk fed to calves in case it reduced their consumption of concentrated feed. Today, this trend is changing due to the availability of a new generation of milk substitutes that maximize the growth of calves from a very young age. An increase in the amount of nutritional composition improved milk replacer fed to calves during the lactation phase has been observed in farms seeking growth above 800 g/day from birth to weaning [30–32]. During this period, the animal undergoes a fundamental change that will endure for the rest of its life: it goes from the monogastric state to the ruminant state through rumen development. Rumen growth is allometric (with growth superior to that of other organs) up to 4 months of age and then is isometric (with growth that is equivalent to that of other organs) [29, 33]. This transition depends on the feeding conditions from birth, as a feeding plan that favors milk intake over concentrate or forages tends to complicate this transition phase toward the ruminant state [29, 34]. Finding a balance between high amounts of liquid-feed intake, starter-grain intake, and rumen development is challenging because of the difficulties involved in providing the correct nutrients. As summarized by Kahn [29], it is important to adequately balance the provision of liquid and solid feed (both from concentrate and roughage) to maximize nutrient intake and gastrointestinal tract development and to minimize postweaning growth reduction [35].

This study aimed to generate a growth differential of at least 200 g/day between two groups of calves and to identify its effect on the onset of puberty. The control (CON) group had a growth-rate target of 800 g/day, whereas the optimized-feeding (OPT) group had a growth-rate target of 1000 g/day from birth to puberty. To create this difference in growth rate, two feeding plans were designed using the US National Research Council (NRC) prediction equations [36]. The 800 g/day feeding plan enables first calving at around 25 months, which already represents an improvement compared with what most breeders do in France today. The second feeding plan of 1000 g/day enables first calving at 22 months, and should define a safety margin for what could be done without altering the animals’ development and health. Regular, standardized measurements of the animals were conducted to determine the impacts of both diets. We used body measurements and quantitative data from automatic feeders, which enabled automated monitoring of each cow’s intakes of milk replacer and solid feed, which improved our understanding of the growth trajectories of individuals. Morphometric measurements such as the thoracic perimeter and the height at withers have long been used, and equations have been developed that enable breeders who do not have a weighing scale to estimate an animal’s weight from these measurements [37]. However, over time morphometric reference values have evolved as a result of genetic selection, i.e., the evolution of animal genotypes.

In addition to aiming to compare the growth rate and age of puberty onset between groups of Holstein dairy heifers that were fed according to one of two feeding plans, this study had the following specific objectives: 1) to monitor changes in morphometric indicators measured from the three major compartments of bone, muscle, and fat; 2) to monitor rumen development; 3) to monitor changes in basic metabolic indicators; 4) and to follow the animals’ global performance from birth to puberty.

## Materials and methods

### Ethical issues

All of the experiments described in this study were carried out in accordance with French National Guidelines concerning the living conditions and welfare of animals used for research purposes, and were performed after being approved by the local ethics committee and the National Department of Research (C2EA No. 19—authorization numbers 201904111810141 and 2019100915307850, APAFiS numbers #20243 and #22355, respectively).

### Animals

Two blocks of Holstein heifer calves were produced through artificial insemination (AI) of the cows and heifers in our research herd (PAO experimental unit, INRAE, Nouzilly, France, Table 1). Table 1 shows the numbers of heifers and cows that were bred to generate the animals used in this study.

**Table 1:** Animals bred to produce the calves of this study.

	Total	Groups—diet^1^		Blocks—season^2^	
		CON	OPT	SUM	WIN
Heifers/cows ratio	22/16	11/8	11/8	13/7	9/9
AI bulls ratio, Lyvolo/Miami	19/19	10/9	9/10	9/11	10/8

^1^Treatment: calves were assigned to control (CON) or optimized-feeding (OPT) groups with average daily gain (ADG) targets of 800 g/day and 1000 g/day, respectively.

^2^Season: SUM = summer; WIN = winter.

The sexed semen used to produce the heifers were from two bulls, “Lyvolo” and “Miami” (Coopérative Evolution, Noyal sur Vilaine, France, Table 1), that were selected for their genetic profiles. At birth, the calves were allocated into two groups according to the following criteria: birth weight, the sire, the dam’s parity, the dam’s genetic score for morphology, the score that was created for ranking bulls according to the French breeding goal (ISU), and the dairy economic index (INEL) that was derived from the dam’s genotyping (Table 2). A first block of 20 females (born from August 12^th^ to September 4^th^, 2019) and a second block of 18 females (born from January 11^th^ to February 16^th^, 2020) were randomized to one of the two treatment groups. The animals were allocated to create two groups of heifers in a way that reduced genetic and experimental bias. Animals in each block were provided the allocated treatment diets (control or optimized) and were followed from birth to puberty.

**Table 2:** Animals bred according to their genetic indices derived from the dam’s genotyping.

	Groups—diet^1^		Blocks—season^2^		SEM^4^	Probability^3^		
	CON	OPT	SUM	WIN		TGE	SE	TGE x SE
ISU index^5^	118	116	117	116	22	0.695	0.861	0.690
Morphology index^6^	0.1	0.3	0.3	0.1	0.8	0.474	0.245	0.531
INEL index^7^	13.6	16.9	17.1	13.1	18.4	0.512	0.477	0.451
Gestation length (d)	279	278	279	278	5.2	0.478	0.608	0.523

^1^Treatment: calves were assigned to control (CON) and optimized (OPT) nutrition groups with average daily gain targets (ADG) of 800 g/day and 1000 g/day, respectively.

^2^Season: SUM = summer; WIN = winter.

^3^Probability of treatment effects: TGE = treatment group effect; SE = season effect; TGE x SE = interaction between treatment group and season effects.

^4^SEM: Greatest value of standard error of the mean within group.

^5^ISU: index for ranking bulls according to the breeding goal (range: 80 to 180).

^6^Morphology score calculating from 30 basic measures mainly related to udder conformation, general bone quality and legs (ranged –2 to +2).

^7^INEL: dairy economic index (ranged –20 °C to +80 °C).

### Housing and feed intakes

At birth, the calves were housed individually in straw-bedded calf hutches (TopCalf, Melesse, France) equipped with individual heating lamps, inside an insulated building that had been specially built for calf rearing. They were fed with their 2 liters of their dam’s colostrum for each of their first two meals: the first meal was given within 2 hours of birth and the second was given within 8 hours of birth. The colostrum was evaluated for quality before administration to the calves using a hand-held digital refractometer (Pal-1—Atago; Marne-la-vallée, France); when the colostrum showed a value below 24 brix, it was supplemented with freeze-dried colostrum (Bonilait Protéines, Chasseneuil du Poitou, France) to reach a value of 24 brix.

Calves were then fed whole milk for two meals per day for 48 hours before switching to a milk replacer (Univor Premium^™^, Bonilait Protéines) at the beginning of the fourth day. To facilitate the transition between whole milk and milk replacer, the meal preceding the introduction of the milk replacer consisted of a rehydrating meal sachet containing mainly sugars, electrolytes and vitamins from groups A, C, D and E (Vitactif^™^ meal pack, Bonilait Protéines). On their fourth day, the animals were transferred to a straw-bedded area meeting European Union standards, where they stayed until they were weaned. The animals had ad libitum access to straw; for bedding, a layer of fresh straw was supplied every day. The two groups were then allocated to different dietary-treatment groups: a CON group following conventional recommended growth targets with an average daily gain (ADG) target from birth to puberty of 800 g/day; and an OPT group with an ADG target from birth to puberty of 1000 g/day.

As the study objective was to follow the heifers’ growth using individual intake measurements that were collected from birth to weaning and from weaning to puberty, our nursery was equipped with an autofeeder with two feeding stations (Alma Pro, Urban, Wüsting, Germany) and automatic concentrate feeders (Hanskamp, Doetinchem, The Netherlands). The animals had access to water ad libitum. Following a specific request to the two above-mentioned companies, the raw data were exported from the feeders. Feeding stations were specially designed so that animals could not enter in groups, so there was no feed stealing. The feeders were recalibrated with each new delivery batch, using procedures specified by manufacturers (10 repetitions). To measure milk-replacer intake (4 meals per day), the volume of feed refused by each heifer was measured by the device at each feed intake and was re-offered to the animal at its next visit. For the solid feed, the feeding plan was designed and split (5 meals per day) as to avoid refusals (4 preliminary trials had been carried out previously). In the rare cases of refusals, the amount of solid feed that was refused was weighed and deducted from the quantity that was reported as having been ingested by the animals. The solid ration consisted solely of dry solid feed, to improve quantitative intake monitoring. Apart from straw, the consumption of which was not measured during this study, the animals had no access to other types of forage.

To maximize our chances of achieving a 200 g/day difference between the two feeding plans while avoiding any bias linked to the qualitative composition of the feed, we chose to base both diets on the same feeds and to change only the amounts of the feeds that were provided (volume and concentration for the milk replacer; weigh for the concentrate solid feed). In total, calves from the CON group each consumed 39.4 kg of milk replacer from day 4 to day 63 (weaning occurred at 9 weeks), whereas calves from the OPT group consumed 59.7 kg of milk replacer during the same time period.

After 2 weeks, calves were given access to concentrate (Floribelle^™^, Tellus, Saint Germain de Salles, France). For the first 7 weeks, the concentrate was not intended to create a difference in growth between the two groups; it was made available to the animals in both groups, in equivalent quantities, to familiarize them with the dry solid feed and facilitate the transition from liquid to solid feed during weaning. As the amount of milk replacer provided was reduced from week 7, the amount of dry solid feed that was provided was increased to become the calves’ primary source of nutrients. As for before weaning, animals in both groups were raised in a straw-bedded area that complied with European standards, the animals had ad libitum access to straw, and fresh straw for bedding was supplied every day. Each calf’s daily ration was split into five meals to avoid feed being left in the troughs. In summary, heifers in the CON group each received 980.1 kg of dry solid concentrate in their first year whereas heifers in the OPT group received 1664.8 kg of dry solid concentrate during the same period.

### Energy and protein formulations of the diets

Food rations were established based on the nutritional values of the feed components (Table 3). At birth calves are not yet ruminants, but they progress to a ruminant stage over the first few months of their development. To compare the two diets during the transition, the nutritional values of the milk replacer and of the solid feed were all converted into metabolizable energy (ME) and apparent digestible proteins (ADP) values using formulas published by the National Research Council in 2001 [36]. Both feeding plans (including milk replacer and solid feed) were designed using NRC prediction equations [36] to allow growths of 800 g/day for the control plan and 1000 g/day for the optimized plan from birth to puberty while providing enough energy and protein to support the desired growth rates without nutrient limitations nor imbalance between energy and protein intake in both diets.

**Table 3:** Nutritional values of the feeds used in this study.

	CMR^1^	Dry solid feed^2^
DM^3^, % of total weight	97	88
CP^4^, g/kg	240	190
ADP^5^, g/kg	223.2^a^	142.5^b^
CF^6^, g/kg	200	23
NDF^7^, g/kg	-	236.2
Ash, g/kg	75.0	68.2
Starch, g/kg	5.0	275.3
ME^8^, Mcal/kg	4.82^c^	2.87^d^

^1^CMR = Calf milk replacer: Univor Premium, Trade mark of Bonilait Protéines, Chasseneuil du Poitou—France.

^2^Dry solid feed: Floribelle, Trade mark of Tellus, Saint Germain de Salles—France.

^3^DM = Dry matter.

^4^CP = Crude protein.

^5^ADP = Apparent digestible protein; ADP = 0.93 x CP for CMR^a^ and ADP = 0.75 x CP for solid feed^b^ [NRC 2001].

^6^CF = Crude fat.

^7^NDF = Neutral detergent fiber

^8^ME = Metabolizable energy; ME = 0.97 x DE with DE = 0.97 GE with GE (Mcal/kg) = 0.057 CP% + 0.092 fat% + 0.0395 lactose% for CMR^c^ (where lactose% = 100—CP%—fat%—ash% [NRC 2001 Eq. 10–5]) and ME = (1.01 x DE—0.45) + 0.0046 (EE-3) [NRC 2001 Eq. 10–8] for solid feed^d^ (where DE = 3.20 Mcal/kg and EE = 23 g/kg as calculated from composition and feedstuffs values [NRC 2001 Table 15–1]).

For the milk replacer, Metabolizable energy (ME) in Mcal/kg DM = 0.97 x Digestible Energy (DE), where DE = 0.97 Gross Energy (GE), where GE in Mcal/kg DM = 0.057 crude proteins in % (CP%) + 0.092 crude fat in % (fat%) + 0.0395 lactose in % (where lactose = 100—CP%—fat%—ash% [NRC 2001 Eq. 10–5]). One kilogram of milk replacer (on a dry matter basis) is therefore equivalent to 4.82 Mcal/kg (Table 3). For solid feed, ME in Mcal/kg DM = 1.01 DE– 0.45) + 0.0046 (EE-3) [NRC 2001 Eq. 10–8], where DE = 3.20 Mcal/kg and EE = 23 g/kg as calculated from composition and feedstuffs values [Table 15–1 in NRC 2001]. One kilogram of solid feed (on a dry matter basis) is therefore equivalent to 2.87 Mcal/kg (Table 3).

To calculate the bioavailability of a heifer’s protein intake, a digestibility coefficient was used to obtain an ADP value. This coefficient depends on the composition of the feed, it applied to crude proteins content, it is 0.93 for milk replacer and 0.75 for solid feed (Table 3) [36, 38].

### Determination of age at puberty onset

To determine the ages of heifers at the onset of puberty, we used two methods in parallel: 1) an assay was performed weekly to measure the progesterone concentration in blood, starting from 5 months of age; and 2) ultrasound was performed weekly to monitor uterine development and ovarian cyclicity. Plasma progesterone concentration was measured by an ELISA assay [39] using 10 μL of undiluted plasma from heifers and primiparous cows; the detection limit of the assay was 0.4 ng/mL, whereas the threshold for determining puberty or any luteal phase was 0.8 ng/mL. For the performance of ultrasonography in the field, we used an Exapad mini ultrasound and a 128-element LR760 transrectal linear probe (IMV imaging, Angoulême, France).

### BW and morphometry

From birth to 12 weeks of age, the animals were weighed once every week using a weighing scale (precision 50 g; Balea, Saint-Mathieu-de-Tréviers, France), after which the weighing frequency was reduced to once every 2 weeks. Each month, seven specific measurements were taken from each animal as follows: 1) the length from the point of the shoulder to the tip of the left ischium (using a seamstress’ measuring tape), 2) the chest circumference at the tip of the withers (using a barymeter, Chambre Régionale d’agriculture de Bretagne, Rennes, France), 3) the height at the withers, 4) the height at the sacrum (using an aluminum rigid gauge, Alliance pastorale, Montmorillon, France), 5) the width at the hips, 6) the width at the external trochanters, and 7) the width at the ischium pins (using a tree caliper Zimmer, Zimming, France). All measurements were recorded via Wi-Fi using a Nomad 800L portable terminal (Agid, Dijon, France).

### Imaging techniques

Ultrasound images were also taken each month using an Exapad mini ultrasound and a 128-element 10 MHz L738P linear probe (IMV imaging; Angoulême, France). They were performed in several bodily locations as previously described [40]: 1) at the buttock at the point of convergence of the facia of the superficial, intermediate, and deep gluteus muscles; 2) at the spine of the 4th lumbar vertebra; and 3) between the 12th and 13th ribs, at the tip of the ribs, with the probe placed vertically.

A total of 12 heifers were given a CT examination at ages 12 and 18 weeks to assess the growth of the rumen and the tibia before animals weighed 200 kg, which is the weight limit supported by the CT table. These examinations were carried out under anesthesia using the following protocol: xylazine (Rompun 2%, Elanco, Germany) was administered by intramuscular injection at a dose of 0.05 mg/kg, and 10 min later butorphanol (Torbugesic Vet 10 mg/ml, Zoetis France, Malakoff, France) was administered by intramuscular injection at a dose of 0.05 mg/kg. With the approval of the ethics committee, the animals were denied access to solid feed on the morning of the CT examination; their last possible meal took place before midnight on the previous day, and the animals were weighed before anesthesia on the morning of the CT examination.

Examinations were carried out by x-ray computerized tomography (Somaton definition AS, Siemens Healthcare SAS, Saint Denis France) using the same x-ray source setting (140 kV, 640 mA/s) for all images to facilitate processing. Raw examinations were performed using Syngo.Via software (Siemens Healthcare SAS, Saint Denis France). All volumes were calculated using the volume tool of the Somaris/7 syngo CT 2012B software package (Siemens Healthineers International, Erlangen, Germany). Bone measurements were taken on the left and right tibias of each heifer. Four ranges in Hounsfield units (HU) were defined to allow the standardized assessment of the volumes of different parts of the bone as follows: total bone (–120 to 2000 HU), cortical bone (800 to 2000 HU), epiphyseal spongy cancellous bone (0 to 800 HU), and medullary cavity (–120 to 0 HU). The volume of the rumen was calculated by selecting pixels within the same range of Hounsfield units for a food bolus (–800 to –10 HU) and for air (–1000 to –800 HU). These assessment ranges were identical for all calves.

### Blood samples

Blood samples were collected from the jugular vein directly into heparinized Vacutainers (Dutcher, Brumath, France) and immediately centrifuged (4500 g for 5 min at 4°C). The separated plasma was stored at −20°C until it was required for assays. The plasma concentrations of triglycerides (Tg), phospholipids (PL), and cholesterol (Chol) were determined by enzymatic assays performed using specific kits from Biolabo SAS (Maizy, France) as follows: triglycerides (reference: LP80519), phospholipids (reference: 99105) and cholesterol (reference: 80106). For each of these assays, the inter- and intra-assay coefficients of variation (CV) were both < 15%. Plasma concentrations of insulin-like growth factor-1 (IGF-1) were determined by ELISA using a specific bovine kit (reference: RAB 1187-1KT) from Sigma Aldrich (Saint-Quentin-Fallavier, France). The intra- and interassay CVs were <10% and <12%, respectively. Non-esterified fatty acids (NEFA) were determined using an enzymatic colorimetry assay (Wako Chemicals GmbH, Neuss, Germany), which had intra- and interassay CVs below 6%.

### Statistical analyses

Statistical analyses were carried out with SAS software (SAS version 9.4, SAS Institute Inc, Cary, NC, USA), and data are presented as means ± SEM. The Gaussian distribution of each measure was assessed by Fisher tests. If the variances were equal, then bi-parametric ANOVA were performed to compare treatment groups, season blocks, and interactions between treatment and season, either at a given age or a given weight. Otherwise, non-parametric Kruskal–Wallis tests and Wilcoxon’s tests were performed. When it was useful to track parameters over time, mixed procedures for repeated effects, including the animal as random effect were used. For all tests, the level of statistical significance was set at P <0.05.

## Results

As birth weight was a criterion that was considered when allocating the calves into the optimized (OPT) and the control (CON) groups, it did not differ between the two groups of calves. However, there was a seasonal effect on this parameter, with summer-born (SUM) calves being lighter than winter-born (WIN) calves (p = 0.020). As is shown in Table 4, indicators related to the energy metabolism of the animals did not differ at birth between the CON and the OPT groups of calves, although calves born in summer showed lower circulating levels of plasma NEFA compared with those born in winter (p = 0.002). In addition, there were no differences at birth between groups or between seasons for plasma cholesterol, phospholipids, triglycerides, or IGF1.

**Table 4:** Plasma indicators of the calves at birth.

Groups	Groups—diet^1^		Blocks—season^2^		SEM^4^	Probability^3^		
	CON	OPT	SUM	WIN		TGE	SE	TGE x SE
Birth weight, kg	38.3	38.2	36.4	**40.3** *	6.5	0.939	**0.020**	0.749
Cholesterol, g/L	2.1	2.0	2.1	2.1	0.4	0.199	0.926	0.946
Phospholipids, g/L	3.1	3.2	3.0	3.2	1.0	0.711	0.515	0.580
Triglycerides, mg/L	178	174	173	179	16.4	0.485	0.207	0.617
NEFA^5^, μmol/L	623	617	597	**646** *	53	0.681	**0.002**	0.825
IGF1^6^, ng/mL	129	130	130	129	5.6	0.348	0.627	0.185

^1^Treatment: calves were assigned to control (CON) and optimized (OPT) nutrition groups with average daily gain (ADG) targets of 800 g/day and 1000 g/day, respectively.

^2^Season: SUM = summer; WIN = winter.

^3^Probability of treatment effects: TGE = treatment group effect; SE = season effect; TGE x SE = interaction between treatment group and season effects.

^4^SEM = Greatest value of standard error of the mean within group.

^5^NEFA = non-esterified fatty acid.

^6^IGF1 = Insulin-like growth factor 1.

*Numbers in bold represent averages that are significantly different with P-value <0.05.

The same CON and OPT lactation plans were followed by animals in the two breeding blocks. The differences that were identified between groups (Table 5) retained significance at each week. There was a difference between blocks in week 1 in favor of the WIN block and in weeks 6 to 9 in favor of the SUM block. Overall, over the nine weeks of the lactation plan, there was a difference between treatments diets (p<0.001) but not between season (p = 0.634).

**Table 5:** Volumes and quantities of milk replacer consumed by calves in this study.

Week	Groups—diet^1^ CMR, kg/week			Blocks—season^2^ CMR, kg/week			Probability^3^		
	CON	OPT	SEM^4^	SUM	WIN	SEM^4^	TGE	SE	TGE x SE
1^5^	1.9	**2.9** *	0.4	2.1	**2.7** *	0.6	**<0.001**	**<0.001**	0.079
2	4.0	**6.4** *	0.3	5.2	5.2	1.3	**<0.001**	0.866	0.146
3	4.8	**7.4** *	0.5	6.2	6.1	1.4	**<0.001**	0.522	0.085
4	5.6	**8.6** *	0.4	7.1	7.1	1.6	**<0.001**	0.553	0.083
5	6.4	**9.5** *	0.7	8.1	7.8	1.7	**<0.001**	0.084	0.131
6	6.3	**9.5** *	0.3	**8.1** *	7.7	1.7	**<0.001**	**<0.001**	0.247
7	4.8	**7.5** *	0.1	**6.2** *	6.0	1.4	**<0.001**	**<0.001**	**<0.001**
8	3.9	**5.7** *	0.2	**5.0** *	4.6	1.0	**<0.001**	**<0.001**	0.118
9^6^	1.6	**2.1** *	0.2	**2.0** *	1.7	0.3	**<0.001**	**<0.001**	0.133
Total	39.4	**59.7** *	1.9	50.0	49.0	2.5	**<0.001**	0.634	0.683

^1^Treatment: calves were assigned to control (CON) and optimized (OPT) nutrition groups with ADG targets of 800 g/day and 1000 g/day, respectively.

^2^Season: SUM = summer; WIN = winter.

^3^Probability of treatment effects: TGE = treatment group effect; SE = season effect; TGE x SE = interaction between treatment group and season effects.

^4^SEM: Greatest value of standard error of the mean within group.

^5^From day 4 to 7 as the milk-replacer feeding plan is beginning on day 4.

^6^Weaning occurred on day 63.

*Numbers in bold represent averages that are significantly different with P-value <0.05.

Up to week 5 after birth, the amount of solid feed consumption was around 100 g/day for each animal, with no differences observed between the two groups (Table 6). From weeks 6 and 7 onwards, feed intake increased without any attempt to differentiate between the animals in the two groups. From week 8 onwards, the feeding plans, and therefore feed consumption, differed significantly between the two groups (p<0.001). There was a difference between SUM and WIN blocks in the first few weeks up to week 8. Then, there was no difference between blocks until week 17. From week 17 to week 26, there was a season effect in favor of block WIN, then between weeks 30 and 36 in favor of block SUM. Overall, cumulative annual consumption differed between the two treatment diet groups (p<0.001) but not between the seasons (p = 0.358). A graph is presented in S1 Fig to illustrate the differences observed between blocks and between groups.

**Table 6:** Quantities of solid feed consumed by calves in this study.

Week	Groups—diet^1^ Solid feed, kg/week			Blocks—season^2^ Solid feed, kg/week			Probability^3^		
	CON	OPT	SEM^4^	SUM	WIN	SEM^4^	TGE	SE	TGE x SE
2–5^5^	0.8	0.8	0.4	**0.9** *	0.6	0.6	0.715	**<0.001**	0.361
6	1.3	1.3	0.5	**1.8** *	1.1	0.4	0.740	**<0.001**	0.400
7	2.7	2.7	0.7	**3.1** *	2.5	1.0	0.727	**0.013**	0.965
8	5.7	**6.2** *	0.4	**6.2** *	5.7	0.6	**<0.001**	**0.001**	0.693
9	8.6	**11.3** *	0.8	10.1	9.8	1.7	**<0.001**	0.101	0.552
10	11.7	**18.4** *	1.9	16.0	14.0	4.1	**<0.001**	0.240	0.410
11	12.7	**23.1** *	0.6	17.8	18.0	5.4	**<0.001**	0.179	0.420
13	15.7	**26.3** *	2.2	20.9	21.1	5.7	**<0.001**	0.975	0.124
17	17.2	**29.1** *	0.9	22.7	**23.7** *	6.3	**<0.001**	**<0.001**	0.141
21	18.5	**34.2** *	0.8	26.1	**26.7** *	8.1	**<0.001**	**0.006**	0.782
26	20.8	**40.3** *	1.6	30.0	**31.2** *	10.3	**<0.001**	**0.002**	0.257
30	22.1	**42.6** *	1.7	**33.2** *	30.8	10.6	**<0.001**	**<0.001**	0.933
36	21.7	**43.5** *	1.6	**32.6** *	32.0	11.3	**<0.001**	**0.015**	0.999
52	29.3	**44.1** *	1.3	36.2	36.9	7.9	**<0.001**	0.425	0.213
Total	980	**1665** *	19.0	1298	1330	360	**<0.001**	0.784	0.958

^1^Treatment: calves were assigned to control (CON) and optimized-feeding (OPT) groups with average daily gain (ADG) targets of 800 g/day and 1000 g/day, respectively.

^2^Season: SUM = summer; WIN = winter.

^3^Probability of treatment effects: TGE = treatment group effect; SE = season effect; TGE x SE = interaction between treatment group and season effects.

^4^SEM: Greatest value of standard error of the mean within group.

^5^No access to solid feed before day 15.

*Numbers in bold represent averages that are significantly different with P-value <0.05.

The amounts of dry matter (DM) intake, ME, and ADP that these quantities of milk and solid feed represent are presented in Table 7. The results are presented according to either age or target weight. The feed intake results were greater in the group of calves that ate the optimized treatment diet for all indicators between all time- and weight-based ranges. From birth to weaning, there was a seasonal effect on the quantities of both milk replacer (p = 0.014) and solid feed (p<0.001) that were consumed. There was also a seasonal effect between 200 and 300 kg in favor of the WIN block, and between 300 and 400 kg in favor of the SUM block. Overall, in terms of cumulative feed intake (i.e., energy and protein) over one year or to reach the target weight of 400 kg, there was a difference between treatments diet groups (p<0.001) but not between seasons (p = 0.173).

**Table 7:** Changes in DM intake, ME intake, and ADP according to age or target weight in both CON and OPT groups^1^.

	Quantity, kg DM^2^			ME^3^, Mcal/kg DM^2^			ADP^4^, kg/kg DM^2^			Probability^5^		
	CON	OPT	SEM^6^	CON	OPT	SEM^6^	CON	OPT	SEM^6^	TGE	SE	TGE x SE
Birth to weaning, CMR^7^	38.2	**57.9** *	1.8	184	**279** *	8.7	8.5	**12.9** *	0.4	**<0.001**	0.634	0.683
Birth to weaning, DR^8^	20.9	**25.1** *	3.4	60.0	**72.0** *	9.8	3.0	**3.6** *	0.5	**<0.001**	**<0.001**	**0.047**
Birth to 6 mo^8^	282	**494** *	8.4	809	**1418** *	24.1	40.3	**70.3** *	1.2	**<0.001**	0.854	0.251
Birth to puberty^8^	766	**869** *	128	2198	**2494** *	367	109	**124** *	18.3	**0.010**	0.307	0.591
Birth to 12 mo^8^	862	**1465** *	16.7	2474	**4205** *	47.9	123	**209** *	2.4	**<0.001**	0.784	0.958
Birth to 100 kg, CMR^7^	38.2	**57.9** *	1.8	184	**279** *	8.7	8.5	**12.9** *	0.4	**<0.001**	0.634	0.683
Birth to 100 kg, DR^8^	**69.4** *	42.6	22.7	**199** *	122	65.1	**9.9** *	6.1	3.2	**<0.001**	0.262	0.367
100kg to 200 kg^8^	307	**361** *	51.9	881	**1036** *	149	43.8	**51.4** *	7.4	**<0.001**	0.103	0.199
200 kg to 300 kg^8^	381	**488** *	65.1	1093	**1401** *	187	54.3	**69.6** *	9.3	**<0.001**	**<0.001**	0.532
300 kg to 400 kg^8^	456	**577** *	59.8	1309	**1656** *	172	65.0	**82.3** *	8.5	**<0.001**	**0.003**	0.589
Birth to 400 kg^8^	1209	**1462** *	121	3470	**4196** *	347	172	**208** *	17.2	**<0.001**	0.173	0.754

^1^Treatment: calves were assigned to control (CON) and optimized-feeding (OPT) groups with average daily gain (ADG) targets of 800 g/day and 1000 g/day, respectively.

^2^DM: Dry matter.

^3^ME: Metabolizable energy.

^4^ADP = Apparent digestible protein.

^5^Probability of treatment effects: TGE = treatment group effect; SE = season effect; TGE x SE = interaction between treatment group and season effects.

^6^SEM: Greatest value of standard error of the mean within group.

^7^CMR: Calf milk replacer.

^8^DR: Dry ration only.

*Numbers in bold represent averages that are significantly different with P-value <0.05.

**Fig 1:**
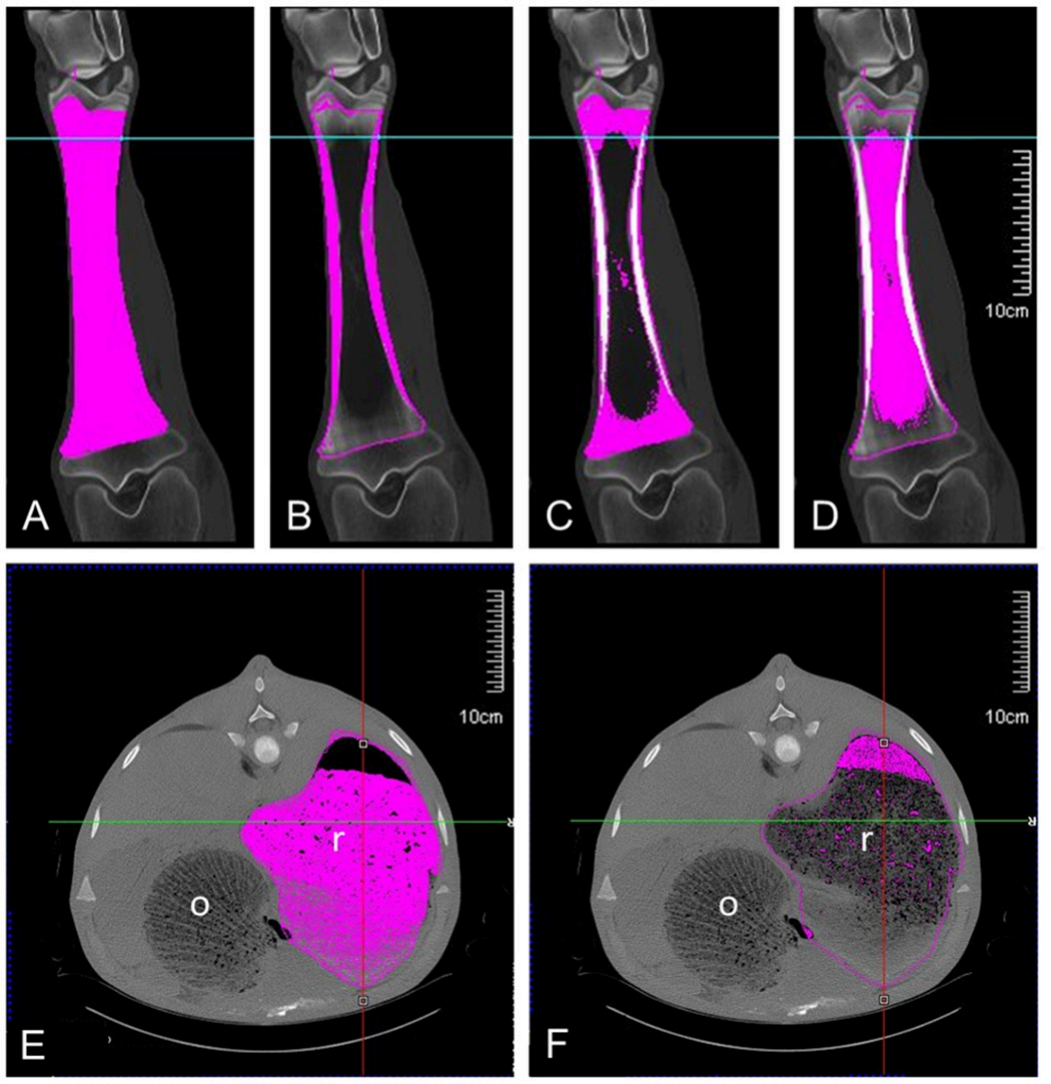
CT scan images of a tibia and a rumen. Photographs showing the different parts of a tibia discriminated by a CT scan with automatic thresholding (A, total bone; B, cortical bone; C, spongy cancellous bone; D, medullary cavity) and of a rumen (E, bolus; F, air) obtained by x-ray CT scanning. The bar represents 10 cm. r, rumen; o, omasum.

As shown in Fig 1A to 1D, we took images of each animals’ tibia bone and its different tissue components using x-ray CT scanning with automatic thresholding.

Calves in the OPT group were heavier than those in the CON group (Table 8) at both 12 and 18 weeks. At 12 weeks of age, the only difference in bone volume found between the groups was in the cortical bone: greater cortical bone thickness was measured in the tibia of calves in the OPT group compared with those in the CON group. At 18 weeks of age, in addition to this difference in cortical bone thickness there was a difference in favor of calves fed with the optimized diet for trabecular cancellous bone. On a per-kilogram-weight basis, cortical bone volume remained greater at 18 weeks of age for OPT calves. At 12 weeks of age, the ruminal mean volumes (Fig 1E and 1F) of the calves in the CON group were higher than those of the calves in the OPT group; this difference in ruminal volume was no longer significant at 18 weeks. All parameters that were assessed changed as a function of time between the two dates, except for medullary cavity volume, which remained constant on a per-kilogram-weight basis.

**Table 8:** Parameters measured in the heifers at the 12-week and 18-week CT assessments.

	Groups—diet^1^							
	12 weeks		18 weeks		SEM^3^	Probability^2^		
	CON	OPT	CON	OPT		TGE	TE	TGE x TE
Weight (kg)	103.5	**123.5** *	136.0	**163.2** *	10.5	**<0.001**	**<0.001**	0.170
Tibia volume MC^4^ (cm^3^)	47.1	48.2	66.7	75.7	16.5	0.209	**<0.001**	0.330
Tibia volume CB^5^ (cm^3^)	42.5	**53.7** *	58.5	**81.9** *	11.8	**<0.001**	**<0.001**	0.130
Tibia volume SCB^6^ (cm^3^)	97.7	111.8	110.5	**139.7** *	23.8	**<0.001**	**0.001**	0.204
Tibia volume MC^4^ (mm^3^/kg)	458	397	491	464	149	0.186	0.133	0.596
Tibia volume CB^5^ (mm^3^/kg)	408	436	430	**502** *	82	**0.002**	**0.006**	0.147
Tibia volume SCB^6^ (mm^3^/kg)	943	906	810	856	167	0.917	**0.027**	0.305
Rumen vol (l)	**14.9** *	9.8	19.2	16.6	3.2	**<0.001**	**<0.001**	0.077

^1^Treatment: calves were assigned to control (CON) and optimized-feeding (OPT) groups with average daily gain (ADG) targets of 800 g/day and 1000 g/day, respectively.

^2^Probability of treatment effects: TGE = treatment group effect; TE = time effect; TGE x TE = interaction between treatment group and time effects.

^3^SEM: Greatest value of standard error of the mean within group.

^4^MC: Medullary cavity.

^5^CB: Cortical bone.

^6^SCB: Spongy cancellous bone.

*Numbers in bold represent averages that are significantly different, with P-value <0.05.

**Fig 2:**
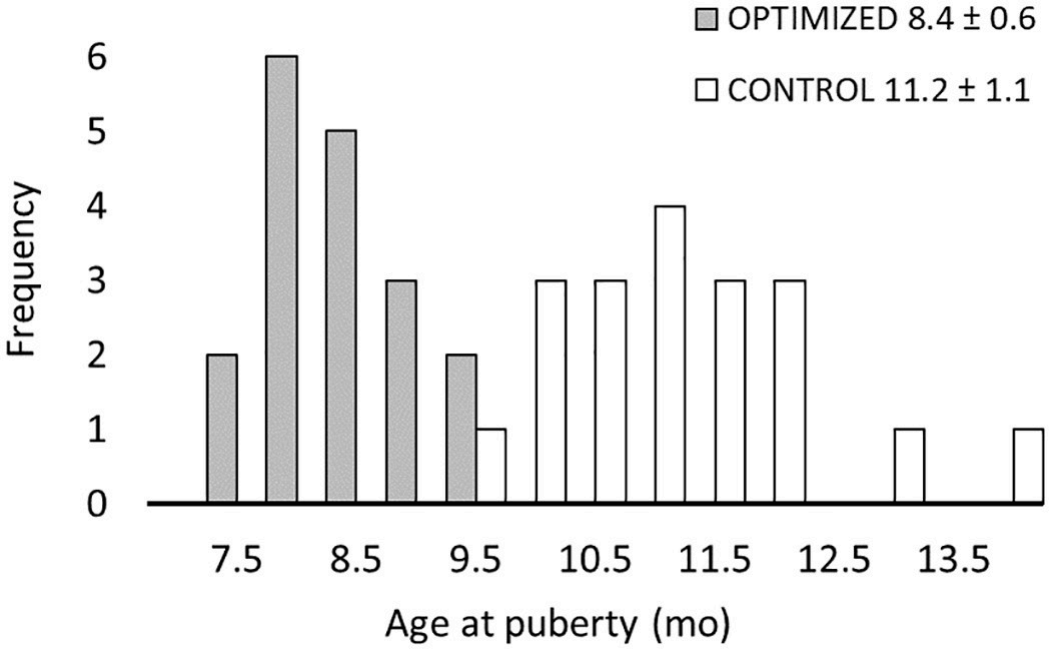
Distribution of the ages in months of heifers at puberty onset. Control (CON) group: white bars, average age at puberty 11.2 ± 1.1 months. Optimized-diet (OPT) group: gray bars, average age at puberty 8.4 ± 0.6 months.

The distribution of animals in the two treatments diet according to the age at puberty with a half-month increment is shown in Fig 2. The age at puberty onset of the OPT calves ranged from 7 months and 18 days to 9 months and 18 days, with an average age of 8.4 ± 0.6 months. In comparison, the age at puberty of the CON calves ranged from 9 months and 12 days to 13 months and 27 days, with an average age of 11.2 ± 1.1 months; this difference in age was statistically significant (p<0.001).

**Fig 3:**
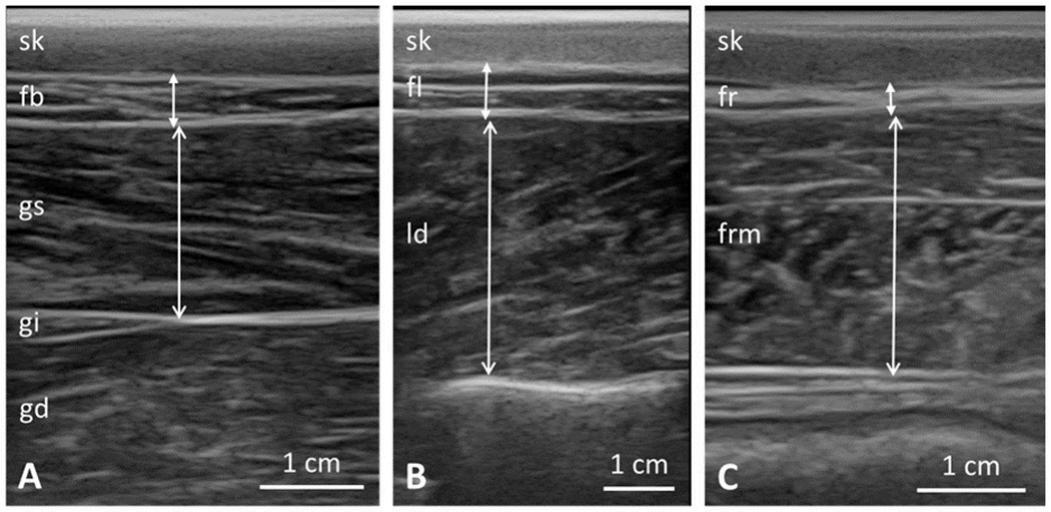
Photographs showing the three locations of ultrasound measurements. Measurements were made at the buttock at the point of convergence of the facia of the superficial, intermediate, and deep gluteus muscles (A); at the spine of the 4th lumbar vertebra (B); and between the 12th and 13th ribs, at the tip of the ribs with the probe placed vertically (C). The bar represents 1 cm in all photographs. Double white arrows and double white open arrows represent the locations where measurements were performed for fat and muscles, respectively. sk: skin, fb: fat thickness at the buttock, gs: gluteus superior thickness, gi: gluteus intermediate, gd: gluteus deep, fl: fat thickness at the lumbar region, ld: longissimus dorsus thickness, fr: fat thickness at the flat rib region, frm: flat rib muscle thickness.

Various measures were selected to describe the heifers’ growth at selected ages (Table 9) or target weights (Table 10). These measures were classified as parameters of general development, skeletal development, development of the muscle or fat compartments (Fig 3), and plasma parameters.

**Table 9:** Changes with age in measurements related to skeletal, muscle, subcutaneous fat, and general growth in both CON and OPT groups^1^.

		Weaning (9 weeks)			6 months			Puberty			12 months		
		CON	OPT	SEM^2^	CON	OPT	SEM^2^	CON	OPT	SEM^2^	CON	OPT	SEM^2^
General development	Age, months	2.2	2.2	0.1	5.6	5.6	0.1	**11.2** *	8.4	1.1	12.1	12.0	0.1
	Weight, kg	81	**95** *	7.8	164	**206** *	12.6	304	298	31.6	324	**403** *	19.6
	ADG^3^, g/day	650	**859** *	87.9	735	**982** *	54.5	780	**1018** *	61.9	779	**994** *	53.3
	TP^4^, cm	101	**103** *	3.8	128	**136** *	3.0	**160** *	155	7.2	164	**174** *	4.6
Skeletal development	Length, cm	90	**94** *	4.0	116	**124** *	6.2	142	142	7.0	145	**154** *	5.2
	HW^5^, cm	91	**95** *	3.3	108	**114** *	2.6	126	124	5.0	128	**133** *	3.3
	HS^6^, cm	96	**100** *	2.6	113	**120** *	2.9	132	131	4.2	134	**140** *	3.5
	WH^7^, cm	22.1	**23.3** *	1.0	29.7	**32.3** *	1.2	39.3	38.5	1.7	40.6	**44.7** *	1.4
	WT^8^, cm	26.1	**27.7** *	1.9	32.3	**35.5** *	1.1	40.7	40.9	1.9	41.8	**45.1** *	1.4
	WI^9^, cm	14.7	**16.4** *	2.0	18.6	**20.8** *	1.3	25.6	24.9	1.7	26.4	**29.1** *	1.1
Muscle comp.	GS^10^, mm	9.5	**11.7** *	1.1	13.0	**15.2** *	1.5	17.3	17.5	1.8	17.5	**19.6** *	1.7
	LD^11^, mm	15.0	**18.1** *	2.2	18.3	**22.4** *	3.4	22.9	23.5	3.5	23.2	**28.1** *	3.3
	FRM^12^, mm	6.4	**7.9** *	1.4	7.6	**9.9** *	1.7	11.4	12.2	2.0	12.0	**15.7** *	2.2
Adipose comp.	FB^13^, mm	0.6	0.6	0.1	0.8	**1.1** *	0.6	1.8	**2.7** *	0.8	2.1	**5.5** *	1.3
	FL^14^, mm	0.8	0.8	0.3	0.9	**1.4** *	0.4	1.8	**2.5** *	0.9	2.3	**4.6** *	1.7
	FR^15^, mm	0.6	0.7	0.3	0.8	**0.9** *	0.2	1.3	**1.6** *	0.5	1.5	**3.6** *	1.4
Plasma parameters	Chol^16^, g/L	2.7	2.7	0.5	2.8	2.9	1.0	2.5	**3.2** *	0.9	2.1	2.0	0.8
	PL^17^, g/L	3.9	4.0	1.1	8.1	8.3	2.1	7.2	8.0	1.8	6.9	6.7	1.2
	Tg^18^, mg/L	179	176	57.1	200	202	27.7	177	207	84.0	200	185	107.8
	NEFA^19^, mmol/L	383	376	38.8	**369** *	343	19.2	**395** *	332	19.1	**404** *	314	16.4
	IGF1^20^, ng/mL	118	**191** *	13.9	144	**215** *	15.7	194	**237** *	18.3	201	**255** *	16.9

^1^Treatment: calves were assigned to control (CON) and optimized-feeding (OPT) groups with average daily gain (ADG) targets of 800 g/day and 1000 g/day, respectively.

^2^SEM: Greatest value of standard error of the mean within group.

^3^ADG: average daily gain from birth;

^4^TP: thoracic perimeter;

^5^HW: height at withers;

^6^HS: height at the sacrum;

^7^WH: width at the hips;

^8^WT: width at trochanters;

^9^WI: width at ischial tuberosity;

^10^GS: gluteus superior thickness;

^11^LD: longissimus dorsus thickness;

^12^FRM: flat rib muscle thickness;

^13^FB: fat thickness at the buttock;

^14^FL: fat thickness at the lumbar region;

^15^FR: fat thickness at the flat rib region;

^16^Chol: cholesterol;

^17^PL: phospholipids;

^18^Tg: triglycerides;

^19^NEFA: non-esterified fatty acids;

^20^IGF1: insulin-like growth factor-1.

*Significantly greater due to feed treatment with P-value <0.05.

**Table 10:** Measurements related to bone, muscle, subcutaneous fat, and general growth made upon reaching target weights in both CON and OPT groups^1^.

		100 kg			200 kg			300 kg			400 kg		
		CON	OPT	SEM^2^	CON	OPT	SEM^2^	CON	OPT	SEM^2^	CON	OPT	SEM^2^
General development	Age, mo	**3.1** *	2.4	0.3	**7.2** *	5.4	0.6	**11.1** *	8.5	1.0	**14.9** *	12.0	1.1
	Weight, kg	102	104	3.6	205	207	7.0	305	306	8.0	401	403	9.8
	ADG^3^, g/day	674	**886** *	80.4	749	**991** *	63.8	778	**1027** *	65.3	804	**993** *	58.9
	TP^4^, cm	108	107	3.9	**138** *	136	3.0	**161** *	157	3.8	**178** *	175	3.7
Skeletal development	Length, cm	99	98	4.8	124	124	4.8	145	143	4.8	156	154	4.7
	HW^5^, cm	96	96	2.6	114	114	3.1	126	125	3.4	134	133	3.0
	HS^6^, cm	101	101	2.5	120	120	2.9	133	132	2.7	140	140	3.6
	WH^7^, cm	24.8	24.5	1.2	33.1	32.4	1.2	39.8	39.4	1.7	44.5	44.5	1.7
	WT^8^, cm	27.7	**28.8** *	1.5	35.2	35.8	1.4	41.1	41.5	1.3	44.7	44.9	1.1
	WI^9^, cm	15.8	16.5	1.2	21.2	21.1	1.2	26.0	25.8	1.4	29.4	28.8	1.6
Muscle comp.	GS^10^, mm	10.7	**12.0** *	1.1	13.9	**15.4** *	1.7	17.1	17.6	1.7	19.2	20.0	2.3
	LD^11^, mm	15.1	**18.5** *	2.5	18.0	**22.3** *	2.4	22.4	23.8	2.8	26.9	28.2	3.6
	FRM^12^, mm	6.5	**8.3** *	1.5	8.5	**9.8** *	1.9	11.3	12.6	2.5	14.1	15.4	2.8
Adipose comp.	FB^13^, mm	0.6	0.7	0.1	0.9	1.2	0.7	1.8	**3.0** *	0.9	3.6	**5.5** *	1.7
	FL^14^, mm	0.8	0.8	0.2	1.2	**1.4** *	0.4	2.1	**3.1** *	1.0	3.3	**4.2** *	1.4
	FR^15^, mm	0.6	0.7	0.3	0.9	1.0	0.3	1.3	**1.8** *	0.5	2.2	**3.2** *	1.4
Plasma parameters	Chol^16^, g/L	2.8	2.9	0.5	2.9	2.7	1.0	2.3	**3.1** *	0.9	2.5	2.5	0.6
	PL^17^, g/L	4.2	4.6	1.1	8.5	7.8	2.0	7.1	**8.3** *	1.4	7.2	7.1	1.3
	Tg^18^, mg/L	185	167	59.2	202	179	60.0	172	206	84.2	193	201	64.7
	NEFA^19^, mmol/L	373	397	44.6	**373** *	345	19.1	**397** *	332	19.9	**409** *	323	17.2
	IGF1^20^, ng/mL	119	**187** *	19.4	159	**209** *	18.2	195	**238** *	20.0	210	**251** *	20.7

^1^Treatment: calves were assigned to control (CON) and optimized-feeding (OPT) groups with average daily gain (ADG) targets of 800 g/day and 1000 g/day, respectively.

^2^SEM: Greatest value of standard error of the mean within group.

^3^ADG: average daily gain from birth;

^4^TP: thoracic perimeter;

^5^HW: height at withers;

^6^HS: height at the sacrum;

^7^WH: width at the hips;

^8^WT: width at trochanters;

^9^WI: width at ischial tuberosity;

^10^GS: gluteus superior thickness;

^11^LD: longissimus dorsus thickness;

^12^FRM: flat rib muscle thickness;

^13^FB: fat thickness at the buttock;

^14^FL: fat thickness at the lumbar region;

^15^FR: fat thickness at the flat rib region;

^16^Chol: cholesterol;

^17^PL: phospholipids;

^18^Tg: triglycerides;

^19^NEFA: non-esterified fatty acids;

^20^IGF1: insulin-like growth factor-1

*Significantly greater due to feed treatment with P-value <0.05.

When results are presented depending on the time, there was a significant difference in favor of the OPT group at 9 weeks, 6 and 12 months for all general and skeletal development indicators as well as for the muscle compartment (Table 9). As expected, ADG was greater for the OPT group at all ages.

At puberty, the indicators of skeletal and muscular development (gluteus, lumbar and flat rib muscles) were not significantly different between the two groups, but the indicators of development of the adipose compartment were higher in the OPT group compared with the CON group from 6 months of age (Table 9). The thoracic perimeter measurements were greater in the OPT group compared with the CON group at ages 9 weeks, 6 months, and 12 months; however, this measure was greater in the CON group at puberty. Finally, among the circulating plasma indicators, NEFA was higher in the CON calves at 6 and 12 months of age and at puberty, whereas IGF1 was higher in the OPT calves at all ages. Note that circulating cholesterol was higher in OPT heifers at puberty.

When the groups were compared according to target weights (Table 10), there was still an advantage for the OPT calves with respect to the width at trochanters and at all three muscle locations at 100 kg. These greater muscular-compartment sizes were maintained at 200 kg; at this weight and in heavier animals, there were greater levels of adipose in the OPT group compared with the CON group. As seen previously at puberty, the thoracic perimeter measurements were higher in the CON group heifers at weights of 200 kg, 300 kg, and 400 kg. As was expected, the ADG was greater for OPT animals at all target weights. Concerning the circulating plasma indicators, we found higher NEFA levels in the CON animals at 200 kg, 300 kg and 400 kg. Finally, we found higher IGF1 levels in the OPT animals at all target weights and higher circulating levels of cholesterol and phospholipids in the OPT animals at 300 kg, i.e., during a period corresponding to puberty.

## Discussion

There was a seasonal effect on the birth weights and the plasma NEFA concentrations of the newborn calves. The phenomenon of the exposure of cows to elevated ambient temperatures during late gestation shortening gestation has already been described by Wright et al. [41], and it results in lighter calves being born during the summer. In summer, reduced daily intake of DM during the last part of gestation, heat stress [42, 43], and/or decreased forage availability may contribute to the lower birth weights of calves [44]. As calf weight positively influences the plasma NEFA at birth [45–47] on one hand, and heat stress is inversely correlated with plasma NEFA concentration at birth on the other hand [48], we expected to see lower NEFA plasma concentrations in the lighter calves that were born in summer [49, 50].

As calf development occurs in the bone and muscle compartments before it occurs in the adipose tissue compartment [51], the first question we wanted to answer was whether the optimized feeding plan enabled earlier mineralization of the skeleton and earlier general body development. An interesting result is that the increased bone development at the cortical level in heifers fed the optimized diet correlated with the early development of bone tissue that later mineralizes. This effect persisted even when the results were normalized to the weights of the animals. At equal weights, the calves fed the optimized diet showed better skeletal development due to the ingestion of the milk replacer almost exclusively for their first 7 weeks, after which they ingested the solid dry feed.

On the contrary, calves of the CON group had greater ruminal development according to the measurements of rumen volume. The rumen volumes of the CON calves are in agreement with what has already been described in the literature, with postweaning rumen volumes of around 14 liters and volumes of 19 liters having been measured at 16 weeks of age [52]. In contrast, the volumes of the rumens of the OPT calves were lower at 12 weeks compare to those of the CON calves. Because the rumen volume increases mainly due to the consumption of fibrous forages [29, 33], we attribute the deficit in rumen development of the calves fed the optimized diet to lower straw intake, even though straw was distributed ad libitum in the present study. It should be noted that smaller rumens do not reflect a functional deficit because the ADG of the OPT calves at this age was on average 1100 g/day, with exceptional calves gaining more than 1300 g/day; at this age, ADG was only around 750 g/day for the CON calves.

The calves on the optimized diet had distinctly earlier ages at the onset of puberty. The oldest age at which puberty was reached in that group was within a few days of puberty onset of the youngest CON calf to reach puberty. Due to their greater nutrient intake and ADG, puberty was achieved on average 3 months earlier in the OPT group compared with the CON group. Puberty does not occur at a fixed age, but rather occurs when the animals have reached a certain level of development. It is common to say that puberty is a function of weight, and this was true in our study as heifers reached puberty at an average weight of about 300 kg. Obviously, it took longer for the heifers in the CON group to reach 300 kg and therefore to reach puberty, but this was also achieved at lower energy and protein costs. What is interesting to note is that as with weight, there were no significant differences between the treatment groups for all other indicators of skeletal and muscular development at puberty. Our data show that puberty can be achieved at an earlier age using the optimized feeding plan, allowing the heifers’ genetic potentials to express themselves. Another characteristic of heifers in the OPT group is that they have a higher fattening rate as early as 6 months of age, i.e., well before puberty, or at equivalent weight compared with animals in the CON group, from 200 kg upward. This raises the question of whether these fat reserves will be an advantage or a disadvantage for the animal’s health, reproduction, and milk production throughout their career on the farm. With an ADG greater than 1000 g/day between the weights of 90 and 320 kg [53, 54] and a solid feed containing 19% crude protein [55], we may have exceeded the recommended levels; time will tell us whether we have impacted the animals’ metabolic balance or influenced their milk production.

The management of feeding of both the control and optimized diets had the expected effects on growth. When the results are presented as a function of time, all indicators greater in the animals that were fed with the OPT plan compare to those feed with the CON plan. Despite the use of the same experimental design and feeding plans in the two blocks of calves, differences corresponding exclusively to minor intake refusals were observed according to the calves’ birth season. Indeed, some calves from the WIN block were affected by a short-lived viral infection between birth and weaning; this episode was responsible for the differences observed in weeks 6 to 9 between the SUM and WIN blocks, for both milk replacer and solid feed. It should be noted that the difference between blocks in week 1 was not due to the viral episode, but rather to the promptness of the calves to go to the teat of the automatic feeder from day 4. These health problems generated slight differences between blocks, which fortunately had no impact on the different feeding treatments in place. Finally, concerning the other differences between the two blocks, sometimes in favor of the SUM block, sometimes in favor of the WIN block, we believe that these are minor differences in growth kinetics around the 300 kg weight target, due to the animals being in summer or winter at different times in their development.

In line with the developmental pattern described by Robelin [51], parameters relating to skeletal and muscular development were initially higher for the calves of the OPT group, followed by those related to adipose tissue. This remained true when animals were compared at an equivalent weight. Among the morphometric indicators measured, trochanter width appeared to be the most sensitive parameter, perhaps because it reflects bone and muscle development. The heifers in the group that benefited from the optimized feeding treatment clearly retained an advantage in terms of muscle development at 100 kg and 200 kg; they lost this advantage at 300 kg (i.e., puberty) and at 400 kg, when their observed advantage switched to indicators reflecting the development of adipose tissue. Some metabolic parameters were also associated with lipid development, such as cholesterol and phospholipids, circulating levels of which are known to be positively correlated with feed intake and white adipose tissue mass [56]. Circulating TG levels were comparable in heifers from both groups. The storage capacity of adipocytes for TG is enormous, resulting in the lowering plasma levels as the volume of adipose tissue expands [57]. Circulating NEFA levels, on the other hand, reflected the mobilization of resources by heifers in the CON group at a time when the animals’ energy requirements were increasing as they approached puberty [58, 59]. Levels of IGF1, which is an important developmental indicator [60–62], were also correlated with feed intake [63, 64]. Furthermore, plasma IGF1 was greater with the high levels of energy and protein in the milk replacer used in the optimized diet [65–67]. Plasma IGF1 concentrations in heifers from the CON group never caught up to those from the optimized diet. This indicates that the somatotropic axis had been the activated from birth in animals fed with the optimized nutritional plan [68, 69].

It is each heifer’s age and weight at breeding, and then age at first calving, that set the growth targets to be aimed for. A heifer’s age at puberty could be considered an indicator of the quality of its growth, as the onset of puberty is more dependent on an animal’s level of development than on its age. We observed that skeletal and muscular morphological parameters were identical in the pubertal heifers of the two groups in this study, irrespective of their age. However, it is costly and time-consuming to determine age at puberty precisely, using progesterone assay or ultrasound, and although research institutes and experimental farms can afford to do this, breeders cannot.

Few studies have made the effort required to simultaneously assess so many zootechnical and metabolic parameters in dairy heifers from birth to one year of age. In the present study, we set out to monitor animal growth by measuring reference indicators for each of the major bone, muscle, and fat compartments, and then to study the interaction of these zootechnical indicators with rumen development and plasma metabolic indicators. Our model does not necessarily correspond to the model that would be chosen by a farmer who does not rely entirely on dry solid feed (the price of which has recently risen sharply), but who seeks to diversify his or her inputs by using any kind of forage available on the farm. On the other hand, this model does have the advantage of being highly analytical, enabling us to accurately follow the growth of individual calves at each stage of development, while also enabling us to calculate the associated costs. In our study, for example, for around 95 US$ extra per calf (90 € as 1 € equals 1.06 US$ today), we produced animals that reached puberty 80 days earlier than normal. If we take into account the advantages of early puberty for the farmer in terms of herd management, in addition to the costs of feeding animals awaiting renewal and the costs of labor and building maintenance, then there is no doubt that the slight extra cost of feeding is offset by what is gained. If we extended the exercise to the 400 kg target weight, a weight that allows heifers to be inseminated, then the extra nutritional costs associated with the optimized diet reached a total of 135 US$ (127 €) per heifer (including milk replacer and solid feed), considering that heifers with the OPT diet will reach this milestone at 12 months, while those with the CON diet will reach this milestone at 15 months on average. Finally, this difference between treatment diets is minimal but these calculations do not include straw consumption by the animals in our study.

To avoid having to economize on calf nutrition while guaranteeing optimal development of the animals, it is necessary to select animal for feed efficiency. Curiously, this trait has changed little over the past 30 years, unlike in other species such as broilers, whose efficiency has improved by 250% in 50 years [70]. Because of their ruminant nature, cattle are destined to be much less efficient than other animal species, as they lose a large part of their energy in ruminal fermentation, and they have a high basal level of energy expenditure due to their large size. Nevertheless, according to Connor [71], selection for improved feed efficiency in growing and lactating dairy heifers by measuring residual feed intake combined with other economically important traits, is certainly a potential way to improve the profitability of milk production. In this study, we saw that the heifers that were fed the optimized diet were all able to reach puberty at 9.5 months of age or earlier. In contrast, the most efficient heifers in the CON group reached puberty at 9.5 months of age, but the control feeding plan allowed the wasteful animals in the CON group to reach puberty much later, with some heifers not reaching puberty until after 13.5 months of age. By combining automated daily weighing with automatic feeders, it would be possible to enable "à la carte" management that is based on each animal’s effective growth rate and achieves optimum growth for all individuals in a population, thereby reducing heterogeneity and maintenance costs.

In conclusion, we created two groups of heifers that were allocated to different feeding plans—optimized versus control—that aimed to achieve first calving at the ages of 21 months (OPT) and 24 months (CON). Compared with the CON group, the heifers in the OPT group showed earlier skeletal mineralization, an increased body fattening and an ADG of 1018 g/day from birth to puberty, which enabled them to reach puberty 3 months earlier; the ADG of the CON heifers was 780 g/day over the same period. Despite the limitations of this study, in which the straw was neither restricted nor measured, this work defines new references for a multitude of morphometric indicators, thus enabling the precise monitoring of Holstein heifer growth from birth to puberty. We will continue to monitor these animals to see whether the advantages gained by the heifers in the OPT group in terms of energy reserves will give them an advantage during their milk production phase, or more generally over their entire careers, without compromising their reproductive potential or health.

## Supporting information

S1 Fig Evolution of body weight according to age in both control (CON) and optimized-feeding (OPT) groups in both summer (SUM) and winter (WIN) blocks. (TIF)
