# Supplementary figures and images for: Multiparameter growth-performance monitoring of Holstein dairy heifers fed on moderate- or high-energy feeding plans from birth to puberty
Source: PLoS One. 2024 Nov 21;19(11):e0314015. doi: 10.1371/journal.pone.0314015 (PMC11581270; doi:10.1371/journal.pone.0314015)

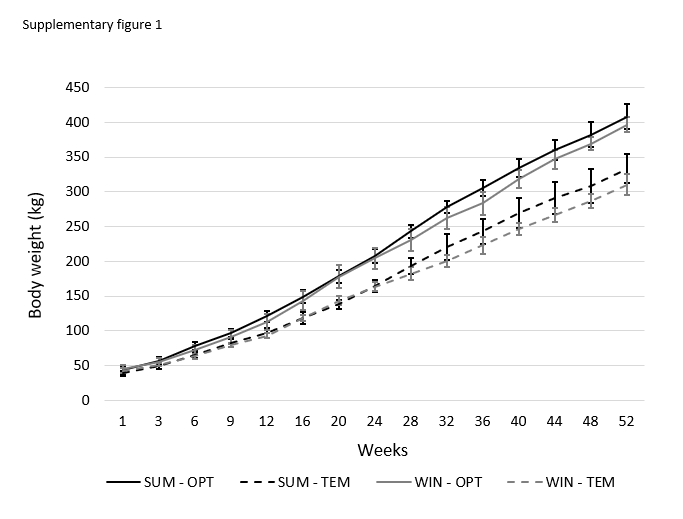

Supplement: S1 Fig — (TIF) [file pone.0314015.s001.tif]
